# Analysis of B cell subsets in severe cutaneous adverse reaction

**DOI:** 10.1186/2045-7022-4-S3-P44

**Published:** 2014-07-18

**Authors:** Hiroaki Azukizawa, Kenichi Kato, Ichiro Katayama

**Affiliations:** 1 Department of Dermatology, Course of Integrated Medicine, Osaka University, Japan; 2 Osaka University, Department of Dermatology, Japan

## Background

T cells play an important role in the pathogenesis of severe cutaneous adverse reaction (SCAR), while the role of B cell immunity in SCAR is unclear. It has been reported that number of B cell and serum IgG are decreased in the patient's peripheral blood of drug-induced hypersensitivity syndrome (DIHS) / drug reaction with eosinophilia and systemic symptoms (DRESS) in the acute stage. Regulatory B cell (Breg) is a IL-10 producing cell and negatively regulates cellular immunity in the mouse autoimmune disease model. Also in the mouse model, B-1 B cell secretes immunoglobulin against microbial infection in a T cell-independent manner. Recently, Breg and B-1 B cell were identified as minor populations of human B cells in the peripheral blood, however, the roles of these B cells in SCAR are unknown. Here, we studied the ratio of Breg and B-1 B cells in the peripheral blood B cells of SCAR patients.

## Method

Three patients of toxic epidermal necrolysis (TEN), DIHS/DRESS, and three healthy controls were involved in this study. Peripheral blood mononuclear cells of SCAR patients in both acute stage and recovery stage were stained. CD24hi, CD27+, CD19+ cells and CD27+, CD43+, CD20+ cells were analyzed as Breg and B-1 B cells, respectively.

## Results

The ratio of B cells in the peripheral blood was decreased in the acute stage SCAR. Furthermore, the ratio of Breg in the CD19+ cells was decreased in the acute stage of DIHS. On the other hand, the ratio of B-1 B cells was different among patients of TEN and DIHS and there was no clear tendency.

## Conclusion

Although the pathological roles of Breg and B-1 B cell in SCAR are still unclear, they might contribute to drug-specific T cell activation or herpes virus reactivation.

